# Effect of buspirone on thermal sensory and pain thresholds in human volunteers

**DOI:** 10.1186/1472-6904-9-12

**Published:** 2009-05-29

**Authors:** Goran Pavlaković, Julija Tigges, Thomas A Crozier

**Affiliations:** 1 Department of Anesthesiology, Emergency and Intensive Care Medicine, University of Göttingen Medical School, Göttingen, Germany

## Abstract

**Background:**

Buspirone is a partial 5-HT_1A _receptor agonist. Animal studies have shown that modulation of serotoninergic transmission at the 5-HT_1A _receptor can induce analgesia in acute pain models. However, no studies have been published so far on the effects of serotonin receptor agonists on pain perception in humans.

**Methods:**

The effects of buspirone (30 mg p.o.) on thermal sensory and pain thresholds were investigated in twelve female volunteers (26 ± 2 yrs) in a prospective, randomized, double-blind, double-dummy, placebo-controlled study with morphine (10 mg i.v.) as positive control.

**Results:**

Morphine significantly increased the heat pain detection threshold (ΔT: placebo 1.0°C and 1.3°C, p < 0.05) at 60 minutes. Buspirone caused mild sedation in six participants at 60 minutes, but was without effect on any of the measured parameters.

**Conclusion:**

Buspirone in the maximal recommended dose was without significant effect on thermal pain. However, as it is only a partial agonist at the 5-HT_1A _receptor and also acts on other receptor types, the negative results of the present study do not rule out a possible analgesic effect of more specific 5-HT_1A _receptor agonists.

## Background

Numerous animal studies have implicated serotonergic pathways, particularly the 5-HT_1a _receptors, in pain perception [1,2]. Serotonin-transporter deficient mice which have lower levels of tissue serotonin have reduced inflammatory pain and thermal hyperalgesia [3]. 5-HT_1a _deficient (knock-out) mice exhibit higher thermal hypersensitivity than naive, non-knock-out mice [4]. The 5-HT_1a _agonist 8-OH-DPAT has analgesic properties in the hot-plate and tail-flick test in mice [5]. Various other 5-HT_1a _agonists, including the partial agonist buspirone, also exhibit analgesic effects in the hot-plate test in mice [6] and in a rat model of surgical pain [7]. Accordingly, 5-HT_1a _receptor antagonists block analgesia induced by stimulation of the lateral hypothalamus [8]. The most likely mechanism is through descending inhibitory pathways that end in the dorsal horn of the spinal cord and affect sensory centripetal neurotransmission.

However, serotonin agonists acting on 5-HT_1a _receptors have also been shown to cause hyperalgesia in rats [9,10], a finding observed by Fasmer et al. with lower doses of buspirone as well [5]. In concordance to these reports, a peripheral injection of 5-HT_1a _agonists produces dose-dependent hyperalgesia, whereas 5-HT_1a _antagonists attenuate serotonin-induced hyperalgesia [11].

5-HT_1a _receptor antagonists, but not 5-HT_1b _receptor antagonists enhance the analgesic effects of tramadol [12], thus providing evidence for an indirect effect of the serotonergic system on descending pain control systems. Similar effects were observed in a recent study which found that 5-HT_1a _antagonism potentiates venlafaxine-mediated analgesia, probably due to blockade of 5-HT_1a _receptors in the raphe nucleus [13]. Furthermore, buspirone inhibited morphine-associated analgesia in rats [14].

These conflicting reports about the involvement of 5-HT_1a _receptors in pain perception in animal studies have been accompanied with only few studies in humans. Hoyer et al. demonstrated that 5-HT_1a _receptors are abundant in the human brain and spinal cord [15]. A recent study showed a correlation of cold pressor pain and 5-HT_1a _receptor binding in the human brain [16], but did not address the question whether a reduction of 5-HT_1a _receptor binding could affect pain perception. Only one study analyzed the effects of buspirone on pain in humans: Kishore-Kumar et al. [17] found that a single oral dose of 20 mg buspirone had no effect on postherpetic neuralgia or painful neuropathy. No human studies on the effects of a pharmacological manipulation of the serotonergic system on acute sensory and pain perception have been published so far.

The present study investigated the effect of buspirone on thermal sensory and pain thresholds in human volunteers.

## Methods

The investigation was designed as a prospective, randomized, double-blind, double-dummy, placebo- and positive-controlled triple cross-over study. The study protocol was approved by the institutional human studies committee of the University of Göttingen and complied with the Declaration of Helsinki on Biomedical Research Involving Human Subjects.

Twelve young, healthy, female volunteers aged 20–30 years were recruited. Exclusion criteria were: pre-existing neurological disease, any acute or chronic drug or alcohol use, diabetes mellitus, pregnancy, known allergy to the tested substances, inability to communicate in the local language and prior wounds or fractures on the tested extremity. Concurrent drug use was ruled out by urine testing on the trial days. Before commencing the study, the subjects were introduced to the study protocol, gave their written informed consent and had one trial thermotesting run prior to the actual measurements.

The study was conducted in three different sessions, seven days apart. On each session, a peripheral venous cannula was inserted and the participants received either 30 mg buspirone p.o. (Bespar^®^, Bristol-Myers Squibb, Germany), 10 mg morphine i.v. (MSI-10^®^, Mundipharma, Germany) in 100 ml saline infused over 15 minutes or placebo. Placebo tablets were prepared by the hospital pharmacy department and were identical to the buspirone tablets. Normal saline (100 ml) was infused over 15 minutes as i.v. placebo. All participants received one tablet and one infusion at each session. At the buspirone session (BUS) they received a buspirone tablet and a dummy infusion of saline, at the morphine session (MOR) they received a dummy placebo tablet and a morphine infusion, and at the placebo session (PLA) they received a placebo tablet and intravenous saline.

### Determination of sensory thresholds

Thermal thresholds were determined five minutes before as well as 60 and 120 minutes after administration of the tested substance, using the TSA-II Sensory analyzer^® ^(Medoc Ltd., Ramat Yishai, Israel) with a skin contact surface area of the thermode of 9 cm^2^. This was attached to the planar side of the forearm of the non-dominant arm by means of an elastic Velcro^® ^tape, taking care to ensure that the entire surface of the thermode was in contact with the skin.

The order in which the test drugs were administered to each participant was randomized in a block design prior to the study. The same investigator, who was blinded to the group allocation, performed all tests.

The basal skin adaptation temperature of the thermode was set to 32°C. After an accommodation period of five minutes at this temperature, the tests were performed using the method of limits, with temperature change of 1°C sec^-1 ^for heat and cold detection and 4°C sec^-1 ^for heat pain and cold pain threshold testing and rapid return to the basal temperature. The more rapid temperature change used to determine pain thresholds was chosen to avoid sensitization of the skin due to the thermal stimuli. All tests were performed in the same quiet room with a constant room temperature of 21°C and always started at 9 a.m. No visual or auditory cues were given to indicate stimulus onset. The sensory qualities were always tested in the same order: cold detection threshold (CDT), cold pain detection threshold (CPDT), heat detection threshold (HDT) and heat pain detection threshold (HPDT). Four measurements were performed for each threshold and the results were averaged. There was a pause of at least ten seconds between each measurement.

Heart rate, blood pressure and Ramsay sedation score (RSS) [18] were assessed before each threshold determination. With the latter, patients are classified on a scale from 1 (anxious and restless) to 6 (no response to stimulus).

### Data analysis

Data are presented as means and standard deviation (unless otherwise stated). Data analysis was carried out with the Statistica program Version 7.1 (StatSoft, Tulsa, OK, USA) in cooperation with our department of biomedical statistics. Thermal sensory and pain detection threshold data were tested for normal distribution with the Kolmogorov-Smirnov test and then analyzed with multiple t-tests for paired data with Tukey-Krame correction factor for multiple comparisons. P-values lower than 0.05 were considered significant.

## Results

The mean age of the participants was 26.5 ± 2.4 years. None of the participants were pregnant, suffered from neurological diseases or were taking any acute or chronic medication. None of the participants dropped out of the study and all drug-screening tests were negative.

**Figure 1:**
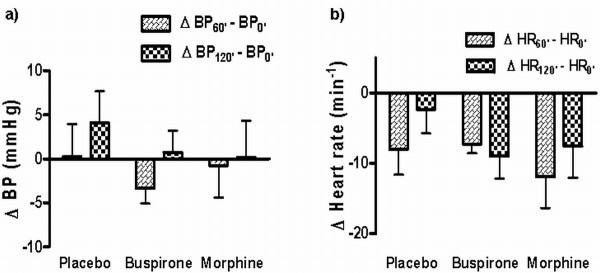
**Hemodynamic parameters**. Hemodynamic parameters 60 and 120 minutes following buspirone and morphine administration: The values are expressed as change in the blood pressure or heart rate from the pre-treatment value. a) Systolic blood pressure change, b) Heart rate change. Blood pressure and heart rate changes following the two drugs were not significantly different than with placebo (p < 0.05). BP_0'_, BP_60'_, BP_120' _= systolic blood pressure at the time of drug administration (0) and 60 and 120 minutes after drug administration. HR_0'_, HR_60'_, HR_120' _= heart rate at the time of drug administration (0) and 60 and 120 minutes after drug administration.

All participants were cooperative and tranquil (Ramsay score 2) at the start of each session. Blood pressure and heart rate were not affected by either of the tested substances at any tested time point (Figure 1).

**Figure 2:**
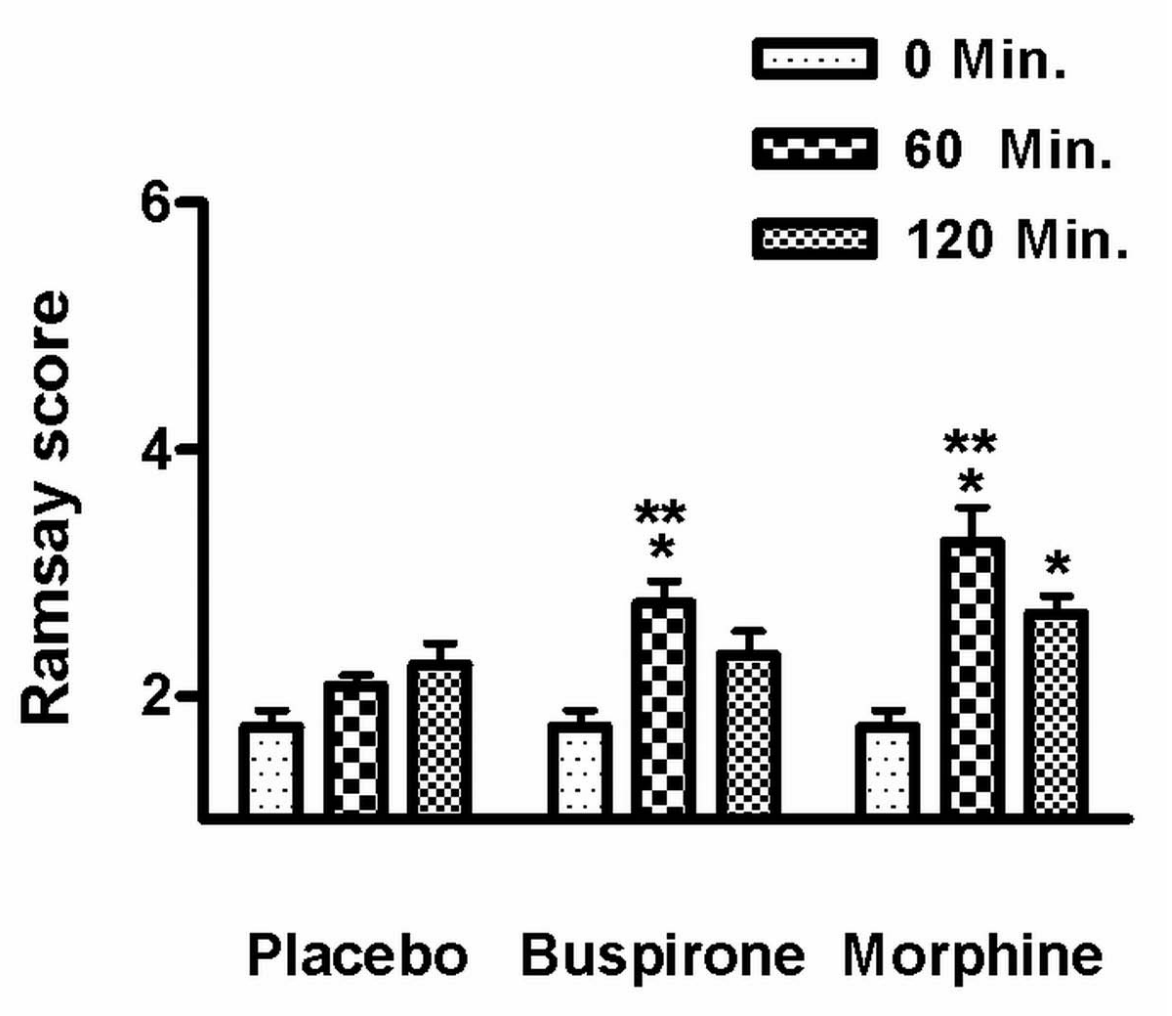
**Sedation scores**. Ramsay sedation scores for the subjects before and 60 and 120 minutes following the drug administration. The initial score always 2 (cooperative and tranquil) in all participants. * = statistical significance of the Ramsay scores compared to pretreatment score (p < 0.05). ** = statistical significance of the Ramsay scores compared to placebo at the same time point (p < 0.05).

Buspirone caused sedation in half of the patients at 60 minutes (Figure 2), but this has largely resolved at 120 minutes. No other side effects were observed or reported. Morphine resulted in a higher incidence and a higher degree of sedation at sixty minutes after drug administration. Four participants complained of dizziness and four of nausea following the infusion of morphine.

**Figure 3:**
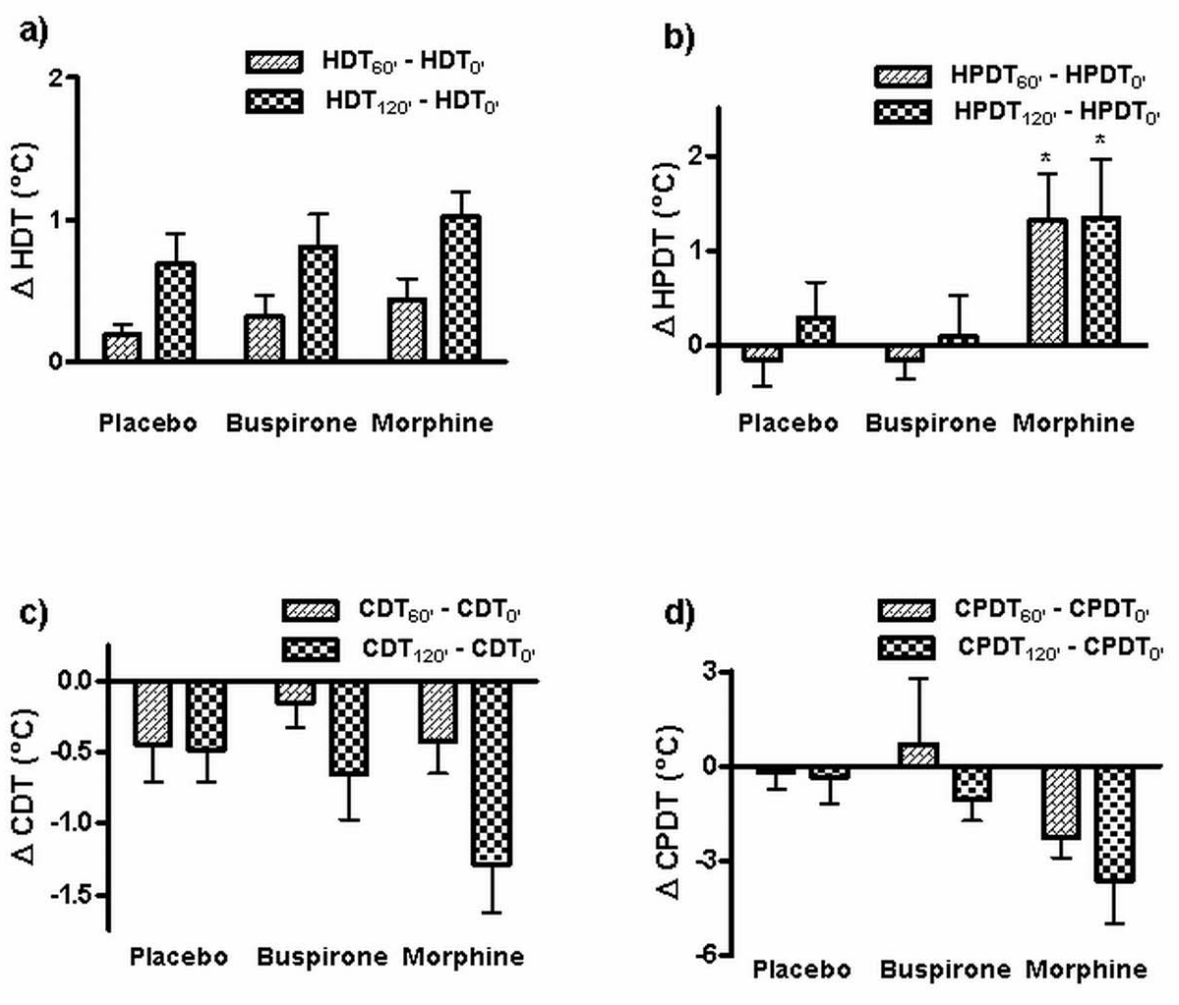
**Thermal sensory and pain thresholds**. The effect of the two drugs (morphine and buspirone) on the tested thermal sensory qualities: a) HDT, b) HPDT, c) CDT and d) CPDT. Data are expressed as means ± SD of the difference between the baseline threshold values measured before and 60 or 120 minutes after the administration. * = statistical significance of change in threshold compared to pretreatment (p < 0.05). HDT = heat detection threshold, HPDT = heat pain detection threshold, CDT = cold detection threshold, CPDT = cold pain detection threshold.

Morphine significantly increased the heat pain detection threshold at the two time points, both compared to baseline as well as placebo (Figure 3). The changes of the thresholds for cold detection and cold pain detection following morphine administration were formally non-significant according to our criteria (p = 0.08 and p = 0.07, respectively, compared to placebo).

Buspirone did not cause a significant change in any of the tested sensory thresholds at either time point after its administration, nor was there any difference compared to the placebo at respective times.

## Discussion

This is the first published study of the effects of a 5-HT_1a _partial agonist on acute thermal sensation and pain perception in human subjects.

A gender homogenous group was deliberately recruited for this study to avoid the documented influence of gender on pain perception and pain reporting, which could have affected data dispersion and complicated data analysis [19,20]. We chose female participants, since the investigator performing the threshold testing was a woman and this could have influenced the response of male participants [21,22].

Morphine induced a significant increase in the heat pain threshold, indicating that the study design was valid and capable of detecting an analgesic effect on heat pain. The changes in the other three sensory thresholds after morphine administration were not statistically significant compared to placebo, however, the calculated p-values of 0.08 and 0.07 for CDT and CPDT, respectively, indicate that this may have been due to insufficient power of the study group size. We found no significant effect of buspirone in the highest recommended clinical dose on the tested thermal thresholds. The reason for this could be due to the pharmacodynamics of buspirone itself or to the details of the study design.

Mico et al. [23] have linked the effects of serotonin on nociception to its action on 5-HT_1A _somatodendritic autoreceptors in the raphe nucleus that control descending inhibitory pathways involved in the control of rostral nociceptive transmission. As described above, buspirone and other 5-HT_1A _receptor agonists can alter the response to pain in experimental animals, but the effects are complicated and depend to some extent on the study design.

Murphy et al. [10] found that stimulation of 5-HT_1A _receptors raised the threshold to thermal pain, but decreased it with regard to mechanical pain. The 5-HT_1A _agonist F13640 was shown to induce both hyperalgesia and analgesia depending on the time after administration and thus possibly depending on the drugs' plasma concentrations [24] with higher concentrations associated with hyperalgesia.

Buspirone was without effect on tail-flick latency in the hot-plate test in rats but significantly attenuated the analgesic effects of morphine [14]. The 5-HT_1A _agonist 8-OH-DPAT similarly attenuated the analgesic effect of acetaminophen in the hot-plate test in mice [25].

The affinity of buspirone is highest for 5-HT_1A _receptors but it is not entirely specific and also acts on other receptors including an antagonistic effect on DA_2 _dopamine receptors. This partial agonistic and somewhat non-specific action on 5-HT_1A _receptors could possibly explain the spectrum of its actions.

Factors unrelated to the relevance of 5-HT_1A _receptors in the modulation of pain perception may also have contributed to the lack of effect in the present study. The temperature change rate of 4°C sec^-1 ^used to determine pain thresholds was chosen in order to reduce sensitization of the skin due to the repeated thermal stimuli, but this may have been too rapid to detect only small alterations of the threshold.

Buspirone has a high hepatic extraction ratio and high first-pass metabolism after oral administration resulting in low bioavailability [26]. While the plasma and CNS concentrations present one hour after administering the maximal recommended dose of 30 mg were adequate to cause sedation, they might not have been sufficient for an analgesic effect. However, should buspirone have analgesic effects at a higher dose, the significant sedative side-effects would probably limit its clinical usefulness. Studies of acute pain in humans using more specific 5-HT_1A _receptor agonists will be required to define their potential use in analgesia or pain modulation.

## Conclusion

This first human study on the effect of a 5-HT_1A _receptor agonist buspirone on acute thermal sensory and pain perception demonstrated that buspirone in a clinically relevant dose has no effect on thermal sensory and pain perception. It does not rule out a possibility that more specific 5-HT_1A _receptor agonists might have analgesic effects.

## Competing interests

The study was funded through the departmental research fund of our institution and was not supported by any company that could profit from the results of the study. The authors declare no financial conflict of interest connected to this study and its results.

## Authors' contributions

GP participated in the conception and design of the study, data analysis and drafting of the manuscript. JT participated in data acquisition and drafting of the manuscript. TAC participated in the conception and design of the study, data analysis and drafting of the manuscript. All authors read and approved the final manuscript.

## Pre-publication history

The pre-publication history for this paper can be accessed here:


